# Relative Bradycardia in a 61-Year-Old Male With Anaplasmosis: A Case Report

**DOI:** 10.7759/cureus.94785

**Published:** 2025-10-17

**Authors:** Jessica A James, Melissa Brown, Samuel M Segal, Maria Gutierrez-Castillo

**Affiliations:** 1 Internal Medicine, Lake Erie College of Osteopathic Medicine, Elmira, USA; 2 Family Medicine, Guthrie Lourdes Hospital, Binghamton, USA; 3 Internal Medicine, Guthrie Lourdes Hospital, Binghamton, USA

**Keywords:** anaplasmosis, fever of unknown origin, heart rate variability, relative bradycardia, tick-borne disease

## Abstract

Human granulocytic anaplasmosis (HGA), or anaplasmosis, is a tick-borne illness caused by *Anaplasma phagocytophilum*, a gram-negative intracellular bacterium. *A. phagocytophilum* is primarily transmitted by *Ixodes scapularis* in the northeast United States and by* Ixodes pacificus* in California. Presenting symptoms typically include fever, chills, malaise, headache, myalgia, and rarely a rash. This case describes a 61-year-old Black male with a complex medical history, including prior tick-borne and arboviral infections (Lyme disease, dengue fever, and chikungunya), hypertension, mixed hyperlipidemia, bilateral carotid artery dissection, gastroesophageal reflux disease, atrial fibrillation with rapid ventricular response, and current tobacco use. This patient presented to an emergency department in upstate New York with a fever, fatigue, constipation, myalgia, and night sweats. Throughout the patient’s hospital course, he maintained a state of relative bradycardia. The patient reported that he had returned from Haiti and the Dominican Republic two weeks prior to presentation in the emergency department and received several mosquito bites while abroad. Initial guideline-based empiric treatment was started with doxycycline due to suspicion of tick-borne illness, given his history of Lyme disease and his onset of symptoms while in upstate New York. Treatment was continued to complete a 14-day course after confirming the diagnosis of anaplasmosis by PCR testing of whole blood. After completing treatment with doxycycline, the patient’s symptoms resolved completely. This case illustrates a unique finding of relative bradycardia and fever of unknown origin in the context of recent international travel and history of tick-borne and arboviral infections.

## Introduction

Anaplasmosis is an acute illness caused by *Anaplasma phagocytophilum*, a gram-negative intracellular bacterium. Anaplasmosis is most commonly transmitted by ticks and has an incubation period of one to two weeks [1]. Formerly known as human granulocytic ehrlichiosis, anaplasmosis primarily affects neutrophils, distinguishing it from human monocytic ehrlichiosis caused by *Ehrlichia chaffeensis* [2]. The incidence of anaplasmosis in the United States has increased more than 25-fold from 273 reported cases in 2000 to 7,280 reported cases in 2023 [3]. Most cases occur in the northeastern and upper midwestern regions of the United States, with the largest number of cases in Minnesota and New York. In New York, anaplasmosis is the second most common tick-borne disease after Lyme disease [4]. 

*A. phagocytophilum* invades neutrophils by binding to P-selectin glycoprotein ligand-1 (PSGL-1), an important component of the leukocyte rolling cascade, the process that allows white blood cells to exit the bloodstream and travel to sites of injury or infection. Once inside a neutrophil, *A. phagocytophilum* blocks lysosomal fusion while contained within its protective phagosome inside neutrophils. By blocking phagosome-lysosome fusion, *A. phagocytophilum* prevents lysosomes from attacking or destroying the virus [5]. Additionally, *A. phagocytophilum* induces a proinflammatory response resulting in neutrophil deactivation and cytokine release, which contributes to tissue injury. The most commonly released cytokines include interleukin-10 (IL-10), interleukin-12 (IL-12), and interferon gamma (IFN-γ). The resulting tissue injury inhibits neutrophils from effectively responding to the infection [1]. 

The most commonly observed symptoms of anaplasmosis include fever, chills, malaise, myalgia, and headache [1,2]. A rash may also be seen in patients coinfected with *Borrelia burgdorferi* [1]. Less commonly, gastrointestinal symptoms, cough, arthralgias, neck stiffness, shock, and organ failure have been observed. The most commonly observed lab abnormalities include thrombocytopenia, leukopenia, elevated C-reactive protein, and transaminitis [1,2]. Additionally, relative bradycardia (RB) has been documented in several patients with anaplasmosis. RB is a finding of growing clinical interest due to the unclear pathophysiologic mechanisms underlying its occurrence in patients with anaplasmosis and other tick-borne illnesses. 

Treatment of anaplasmosis involves antibiotic therapy, usually doxycycline 100 mg orally twice daily for 14-21 days [1]. In pediatric and pregnant patients, rifampin is a commonly used alternative to doxycycline [2].

## Case presentation

A Black 61-year-old male presented to an emergency department in upstate New York with complaints of fever (maximum temperature of 103.5°F reported before admission), fatigue, constipation, myalgia, and night sweats for the past three days. The patient’s past medical history included hypertension, mixed hyperlipidemia, bilateral carotid artery dissection, gastroesophageal reflux disease, atrial fibrillation with rapid ventricular response, and current tobacco use. His home medications consisted of amlodipine, aspirin, and omeprazole. He did not take any medications for heart rate control. Two weeks prior to his presentation in the emergency department, he had returned from a trip visiting his family in Haiti and the Dominican Republic, where he was bitten by mosquitoes multiple times. He reported a history of Lyme disease, dengue fever, and chikungunya. He denied any recent contact with ill individuals, as well as symptoms such as cough, congestion, pain, or dyspnea. His temperature on arrival at the emergency department was 102°F, and his heart rate was 83 beats per minute. Laboratory findings from the day of admission indicated leukopenia and elevated creatinine phosphokinase (CPK), which was greater than five times the upper limit of normal, meeting the criteria for rhabdomyolysis. Other pertinent laboratory results included creatinine, C-reactive protein (CRP), and ferritin (Table 1).

**Table 1:** Pertinent laboratory findings

Test (units)	Reference range	Day 1 (admission)	Day 2	Day 3	Day 4
White blood cells count (K/uL)	4.5-11	3.1	3.33	4.88	6.71
Hemoglobin (g/dL)	13.5-17.5	14.3	12.7	12.1	12.9
Hematocrit (%)	41-53	42.3	37.3	36.3	39.9
Mean corpuscular volume (FL)	80-100	77.5	77.4	78.4	78.9
Mean corpuscular hemoglobin (PG)	25-35	26.2	26.3	26.1	25.5
Platelets (K/uL)	163-337	191	166	158	189
Creatinine phosphokinase (U/L)	25-90	1,497	1,209	567	291
Creatinine (mg/dL)	0.6-1.2	1.81	1.20	0.91	0.87
C-reactive protein (mg/dL)	<1.0	7.23	6.71	-	6.05
Ferritin (ng/mL)	20-250	-	-	-	508.8

A comprehensive respiratory panel was negative for viral respiratory infections. A peripheral blood smear showed no evidence of intracellular parasites. Lyme disease antibody testing was positive for IgG antibodies and negative for IgM antibodies, confirming the patient’s reported history of Lyme disease. Polymerase chain reaction (PCR) testing was negative for *Ehrlichia chaffeensis*, *Babesia microti*, and *Borrelia miyamotoi*. However, PCR testing for *Anaplasmosis phagocytophilum* was positive, confirming a diagnosis of anaplasmosis. Given his recent travel history and prior arboviral infections, antibody testing for dengue and chikungunya viruses was ordered. Both tests returned positive for IgG antibodies and negative for IgM antibodies. This allowed us to rule out acute chikungunya virus. Given the positive anaplasmosis PCR test and significant improvement on doxycycline, further testing to completely rule out acute dengue virus was not pursued. Testing for the Zika virus showed negative results for IgM and IgG antibodies. 

With the diagnosis of anaplasmosis established, the inpatient infectious disease team was consulted. The patient was continued on doxycycline 100 mg twice daily for 14 days. An abdominal CT scan showed evidence of a large stool collection and a possible stool ball; however, an occult parasitic infection could not be ruled out. Therefore, a one-time dose of albendazole 400 mg for possible helminthic coinfection was given. He was also given 2 liters of intravenous normal saline on admission day 1. The patient's leukopenia and rhabdomyolysis resolved during his hospital stay. In addition, his CRP began downtrending by day 2 of admission (Table 1). The patient reported rapid improvement in his symptoms while in the hospital, and he was discharged on day 4 of his hospital stay. By the time he finished his 14-day course of doxycycline, all of his symptoms had resolved completely.

## Discussion

A primary focus of this case report is the presentation of relative bradycardia (RB) in a patient infected with *A. phagocytophilum*. RB is a symptom of growing clinical interest and may be helpful in differentiating infectious and noninfectious disease states. RB describes a state of dissociation between temperature and heart rate [6]. Patients exhibiting RB do not show the expected physiologic rise of 8-10 beats per minute for each degree of increase above 101°F [6,7]. For example, a fever of 101°F correlates with 108-110 beats per minute, and 103°F correlates with 124 to 130 beats per minute [7]. In this patient, the most dramatic instance of RB documented was a heart rate of 88 beats per minute with a temperature of 102.9°F. While RB has been previously reported in anaplasmosis [8,9], this phenomenon remains underrecognized, and its underlying mechanisms are not fully understood. 

Heart rate variability (HRV), a measure of beat-to-beat fluctuations, has been proposed as a predictor for relative bradycardia [6]. Healthy individuals exhibit physiologic heart rate variability, which increases during inspiration and decreases during expiration [10]. During inspiration, diaphragmatic contraction creates negative intrathoracic pressure, enhancing venous return to the right atrium. This increased preload stretches cardiac myocytes and increases heart rate, a phenomenon known as the Bainbridge (atrial) reflex [11]. In the context of intracellular infections like anaplasmosis, disruption of autonomic signaling through varied mechanisms may impair HRV and contribute to the development of RB. In our patient, HRV was not measured because the presence of previously diagnosed atrial fibrillation would have confounded any measurements due to the inherently irregular R-R intervals.

Studies have shown that dysregulation of several mediators, including nitric oxide, tumor necrosis factor-alpha (TNF-α), interleukin-6 (IL-6), endothelin-1, and nicotinamide adenine dinucleotide phosphate (NADPH) oxidase, can blunt the normal sympathetic response to infection, leading to increased parasympathetic tone and a paradoxically low heart rate despite fever [6]. Studies have shown that *A. phagocytophilum* stimulates the production of several proinflammatory cytokines, including IL-1β, IL-6, IL-8, IL-10, TNF-α, and interferon-gamma (IFN-γ), which contribute to neutrophil recruitment and tissue injury [12,13]. Notably, IFN-γ, which is elevated in anaplasmosis, has also been associated with RB in systemic lupus erythematosus [14]. Similarly, elevated IL-6 and TNF-α levels were reported in a case of cyclic neutropenia associated with RB [15]. However, these data remain correlational; despite a compelling theoretical link, causation between cytokine elevation and RB in anaplasmosis has not yet been established. Additionally, cytokine levels were not measured in this patient. Nevertheless, existing literature supports a role for inflammation-driven autonomic dysregulation in the development of RB. Further research is needed to clarify the pathophysiology underlying this phenomenon.

## Conclusions

This case highlights the presentation of relative bradycardia (RB) in a patient with anaplasmosis. With prompt diagnosis and treatment, the patient made a complete recovery and remained asymptomatic, as verified by a follow-up phone call with the patient one month after his initial presentation.

Recognizing the frequent occurrence of RB in patients with anaplasmosis may prompt further research into the pathophysiological mechanisms of RB in anaplasmosis infection. Future research endeavors should investigate how RB and other clinical manifestations vary among patients infected with anaplasmosis. Specifically, longitudinal studies that monitor heart rate variability and cytokine profiles throughout the progression of the illness and its treatment may provide insight into the involvement of the autonomic nervous system and immune system. Gaining a deeper understanding of these underlying processes could facilitate earlier recognition of these diseases and improve the overall clinical management of tick-borne febrile illnesses, ultimately improving patient outcomes.
